# Antioxidant and Toxic Activity of *Helichrysum arenarium* (L.) Moench and *Helichrysum italicum* (Roth) G. Don Essential Oils and Extracts

**DOI:** 10.3390/molecules27041311

**Published:** 2022-02-15

**Authors:** Asta Judzentiene, Jurga Budiene, Irena Nedveckyte, Rasa Garjonyte

**Affiliations:** 1 Department of Organic Chemistry, Center for Physical Sciences and Technology, Sauletekio Avenue 3, LT-10257 Vilnius, Lithuania; jurga.budiene@ftmc.lt (J.B.); rasa.garjonyte@ftmc.lt (R.G.); 2 Institute of Biosciences, Life Sciences Center, Vilnius University, Sauletekio Avenue 7, LT-10257 Vilnius, Lithuania; irena.nedveckyte@gf.vu.lt

**Keywords:** *Asteraceae*, *Helichrysum arenarium* (L.) Moench, *Helichrysum italicum* (Roth) G. Don, essential oil composition, GC-MS, methanolic extract composition, HPLC-DAD-TOF, DPPH^●^ and ABTS^●+^ tests, cyclic and square wave voltammetry, in vivo toxicity

## Abstract

*Helichrysum arenarium* (L.) Moench (sandy everlasting) is the only species from genus *Helichrysum* Mill that grows spontaneously in Lithuania. The chemical composition of the essential oils (EOs) from inflorescences and leaves of *H. arenarium* wild plants was analysed by GC-MS. Palmitic (≤23.8%), myristic (≤14.9%) and lauric (6.1%) acids, *n*-nonanal (10.4%), and trans-β-caryophyllene (≤6.5%) were the major constituents in the EOs. For comparison, the main components in EO from flowers (commercial herb material) of *H.* *italicum* were γ-curcumene (21.5%), β-selinene (13.6%), α-selinene (8.1%), β-eudesmol (8.3%), and α-pinene (6.5%). Composition of *H. arenarium* methanolic extracts was investigated by HPLC-DAD-TOF. The main compounds were the following: luteolin-7-*O*-glucoside, naringenin and its glucoside, apigenin, chlorogenic acid, arenol, and arzanol. Antioxidant activity of EOs and extracts was tested by DPPH^●^ and ABTS^●+^ assays. Sandy everlasting extracts exhibited significantly higher radical scavenging activities (for leaves 11.18 to 19.13 and for inflorescences 1.96 to 6.13 mmol/L TROLOX equivalent) compared to those of all tested EOs (0.25 to 0.46 mmol/L TROLOX equivalent). Antioxidant activity, assayed electrochemically by cyclic and square wave voltammetry correlated with total polyphenolic content in extracts and radical scavenging properties of EOs and extracts. The toxic activity of EOs of both *Helichrysum* species was evaluated using a brine shrimp (*Artemia salina*) bioassay. *H. italicum* inflorescence EO was found to be toxic (LC_50_ = 15.99 µg/mL) as well as that of *H. arenarium* (LC_50_ ≤ 23.42 µg/mL) oils.

## 1. Introduction

The genus *Helichrysum* Mill (sect. *Stoechadina*, tribe *Gnaphalieae*) containing approximately 600 species is distributed throughout the entire world. The *Helichrysum* species have been used in many folk medicines, as flavouring spices or as food supplements, and for ornamental, cosmetic, and pharmaceutical purposes as well.

*H. italicum* (Roth) G. Don is a typical endemic Mediterranean species, subdivided into several subspecies, such as *italicum*; *michrophyllum* (Willd.) Nyman; *picardii* (Boiss & Reuter) Franco; *pseudolitoreum* (Fiori) Bacch. & al.; *serotinum* (Boiss) P. Fourn; and *siculum* (Jord. & Fourr.) Galbany, L. Sáez & Benedí [1,2,3] and herein cited.

Most of the reports concerning the volatile chemistry of the genus *Helichrysum* have described the composition of essential oils (EOs) of Mediterranean taxa. Variability in chemical composition of *H. italicum* EOs and other extracts is highly affected by genetic factors, phenological stage of development, differences in eco-climatic characteristic of growing habitats, and extract preparation techniques, as reviewed by Maksimović et al. [4] and references therein. Recent data on major chemical composition of *H. italicum* (Italian immortelle) EOs are provided in Supplementary Materials (Table S1) [5,6,7,8,9,10,11,12,13,14,15,16,17,18,19,20,21,22,23,24,25,26,27,28,29,30,31,32,33,34,35]. Numerous research works have been focused on evaluation of various bioactivities of plant extracts [3] and herein cited. EOs or other extracts of *H. italicum* plants exhibited antimicrobial and antifungal [15,21,22,24,27,32,33,35,36,37,38,39,40,41] and herein cited, antioxidant [17,18,27,30,33,34,35,39,42,43,44,45], wound healing and anti-inflammatory [23,35,43,46], anti-proliferative [24], anti-hyperglycaemic [35], acetylcholinesterase inhibition [30], anticancer [34], anti-HIV [46], anti-collagenase, and anti-elastase activities [28]. Additionally, depending on insect species, *H. italicum* EOs exhibited repellent [47] or anti-repellent [9] or larvicidal properties [7]. Strong phytotoxic activity of Italian immortelle EOs against germination of radish and garden cress has been determined [10].

*Helichrysum arenarium* (L.) Moench is a herbaceous perennial plant naturally distributed in Central, Eastern and South-Eastern Europe, North of Balkans, West Siberia, Central Asia, Mongolia, and China [48,49]. *H. arenarium (*also named mainly as sandy everlasting) is the only species from genus *Helichrysum* that grows spontaneously in Lithuania. It is a small (15–40 cm) semi-rosette shrub with flowers of varying colour intensities, ranging from citric-yellow to yellow-brown, dark-orange, or even orange-brown. The plants with orange inflorescences are attributed to the morphological form f. *aurantiacum* (Pres.) Bleck. Other two forms—f. *compactum* Beckhaus and f. *divaricato-ramosum* Abromeit—found in Lithuania are rare [48]. The most suitable ecosystems for *H. arenarium* are forests and meadows in dry, poor sandy soils. Natural sources of the plant are rather limited in Lithuania; therefore, harvesting the sandy everlasting from wild habitats is limited as it can increase impoverishment or even extermination of population of the species. For these reasons, *H. arenarium* is under protection in many European countries.

The inflorescences of *H. arenarium* (*Helichrysi flos*), being rich in flavonoids, essential oils, fatty acids, carotenoids, phytosterols, bitter substances, phenolic compounds, vitamins, and mineral salts, have been used in European herbal medicine for its choleretic, cholagogue, diuretic, anti-inflammatory, hepatoprotective, digestive, anti-atherosclerotic, detoxifying, antimicrobial, antioxidant, cytogenetic, anti-cancer, and anti-hyperglycaemic properties [49,50,51,52,53,54,55,56,57,58,59,60,61,62,63,64,65,66,67,68]. *Helichrysi flos* is used in Lithuanian folk medicine for healing gall-bladder and gastric disorders, cystitis, and arthritis. The dried plant is applied as a repellent against brown house moths *Hofmannophila pseudospretella* (Stnt.) (Lepidoptera, Oecophoridae).

Investigations dealing with *H. arenarium* plant extracts have focused mostly on non-volatile phytochemicals [45,49,50,57,58,59,60,61,62,63,65,66,67,68,69,70,71,72,73,74,75,76,77], while only 11 available papers concerning sandy everlasting volatiles have been published over the last 20 years [56,62,64,74,78,79,80,81,82,83,84]. A limited number of studies on EOs properties was devoted mainly to antimicrobial and antifungal activity [56,62,64]. For details related to *H. arenarium* EOs composition and properties, see Table S2 in Supplementary Materials [56,62,64,74,78,79,80,81,82,83,84].

The aim of this work is: (i) to evaluate differences in the chemical composition of EOs of *H. arenarium* and *H. italicum*; (ii) to investigate and compare antioxidant activity of EOs and methanolic extracts by ordinary DPPH^●^ and ABTS^●+^ (TROLOX equivalent) assays and by means of cyclic and square wave voltammetry; (iii) to present composition of sandy everlasting methanolic extracts; iv) to examine in vivo toxic properties of EOs and extracts of both *Helichrysum* species by the *Artemia salina* larvae lethality test.

## 2. Results

### 2.1. Chemical Composition of Essential Oils

The main composition of *H. arenarium* (inflorescences and leaves) and *H. italicum* (inflorescences) EOs is presented in Table 1. Each investigated oil contained up to eight major (with quantity over 3.0%) constituents. Palmitic (hexadecanoic, 23.8 ± 1.13%), myristic (tetradecanoic, 14.9 ± 1.05%), and lauric (dodecanoic, 6.1 ± 1.35%) acids were three main constituents in *H. arenarium* inflorescence oil. This oil contained appreciable amounts of terpenoids trans-β-caryophyllene (5.4 ± 0.55%) and phytone (hexahydrofarnesyl acetone) (4.4 ± 0.55%). Palmitic acid (18.8 ± 0.70%), *n*-nonanal (10.4 ± 1.50%), and myristic acid (8.7 ± 1.35%) were the main compounds in the *H. arenarium* leaf EO. The latter oil contained remarkable quantities of trans-β-caryophyllene (6.5 ± 0.55%), α-pinene (4.2 ± 1.15%), and 1,8-cineole (3.9 ± 0.60%). Inflorescence EO of commercial *H. italicum* was characterized by sesquiterpene hydrocarbons: γ-curcumene (21.5 ± 2.50%), β-selinene (13.6 ± 1.65%), β-eudesmol (8.3 ± 0.35%), α-selinene (8.1 ± 0.55%), and α-pinene (6.5 ± 1.50).

Detailed chemical composition of *H. arenarium* and *H. italicum* EOs is given in Table S3 of Supplementary Materials. Fifty-three and fifty-five identified constituents comprised 96.4 ± 1.52% and 99.1 ± 0.44% of the total amounts of *H. arenarium* inflorescence and leaf oils, respectively. Thirty-two identified compounds comprised 89.4 ± 0.15% of total Italian immortelle EO.

### 2.2. Chemical Composition of Methanolic Extracts

Up to 29 compounds were identified tentatively in *H. arenarium* inflorescence and leaf extracts (Table 2). Due to numerous detailed publications on *H. italicum* extracts [3,4,41,43,44,45] and herein cited, the composition of extracts of Italian immortelle commercial herbs was not investigated in the present study**.**

### 2.3. Total Phenolic Content

Total phenolic content was about 1.6-fold higher in the *H. arenarium* leaf methanolic extract (1062.82 ± 12.36 mg/L of gallic acid equivalent), compared to that in flower (652.56 ± 5.87 mg/L) extract.

### 2.4. Antioxidant Activity Tests

#### 2.4.1. Spectrophotometric (DPPH^●^ and ABTS^●+^) Assays

The antioxidant activity of *H. arenarium* and *H. italicum* EOs and *H. arenarium* extracts tested by DPPH^●^ assay is presented in Table 3. The activity of EOs was similar, practically not varying with species, plant organs, and year of raw material collection. A significant difference was observed between sandy everlasting methanolic extracts and EOs and between methanolic extracts themselves. The activity of leaf extract (19.13 ± 0.04 mmol/L, TROLOX equivalent) was three-fold higher compared to that of inflorescence extract (6.13 ± 0.04 mmol/L).

ABTS^●+^ assay data showed the same tendency as the DPPH^●^ results: activity of sandy everlasting EOs was similar as well, and activity of methanolic leaf extracts (11.18 ± 0.002 mmol/L, TROLOX equivalent) was also significantly higher (about 6-fold), compared to that of flowers (1.96 ± 0.01 mmol/L) (Table 4).

#### 2.4.2. Electrochemical (Cyclic and Square Wave Voltammetry) Assays

Electrochemical techniques cyclic and square wave voltammetry (differing in modes of potential application to the working electrode) are widely used to obtain information about redox active substances in solutions [86]. The working metal or carbon-based electrode is immersed in the sample and its potential scanned to the positive direction. During this forward scan, the potential of the working electrode gradually becomes more positive and, therefore, the oxidizing power of the electrode increases. As soon as the potential of the electrode reaches the oxidation potential of the electroactive sample constituent, oxidation of the compound occurs: the lower the potential of oxidation, the more powerful the reducing, i.e., antioxidant, properties of the compound. The oxidation (anodic) peak potential value (Epa) depends on the chemical structure of the electroactive substance, electrode material, composition, and pH value of the solution. The magnitude of the oxidation (anodic) peak current (Ipa) at Epa is related to the concentration of the electroactive compound. During the reverse scan, reduction currents are registered. The presence of reduction (cathodic) peaks Ipc at reduction (cathodic) potentials Epc in the reverse scan provides information about the reversibility of the redox reaction of the oxidized compounds generated in the forward scan.

Low-cost chemically inert carbon paste electrodes are simply prepared from graphite powder and a liquid inert binder. Carbon paste electrodes have more advantages, such as low background currents, rapid renewal of the surface, and easy modification [87].

To test the antioxidant properties of EOs obtained from inflorescences *H. italicum,* and inflorescences and leaves of *H. arenarium,* a strategy of modification of bulk carbon paste with these oils was used. Our former investigation of EO isolated from *Eupatorium cannabinum* [88] has shown that this approach is suitable to detect easily oxidizable (E_pa_ in the region 0.04 to 0.1 V) compounds, i.e., possible antioxidants, by means of cyclic or square wave voltammetry. However, in the case of investigated EOs from both *Helichrysum* species, cyclic or square wave voltammograms (not shown) did not reveal any oxidation currents throughout the potential region—0.2 to 1.0 V.

**Figure 1:**
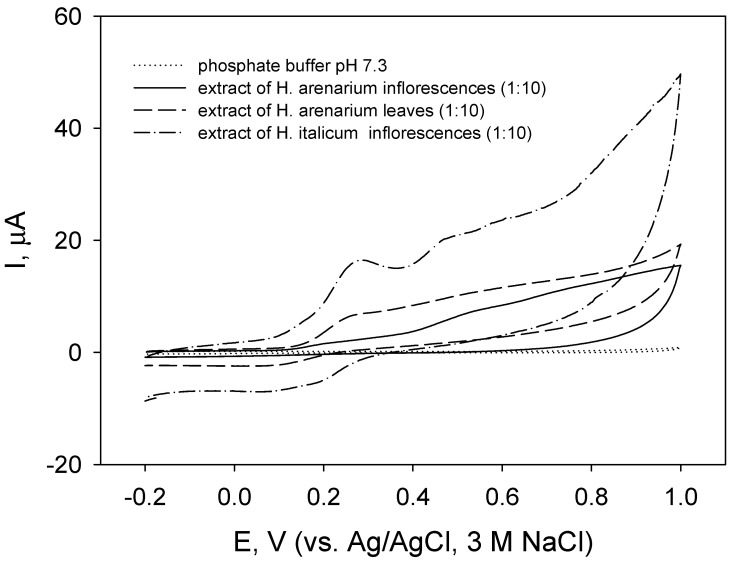
Cyclic voltammograms of carbon paste electrodes in extracts inflorescences and leaves of *H. arenarium* and inflorescences of *H. italicum* (as indicated) in phosphate buffer pH 7.3, potential scan rate 100 mV/s.

Contrary to the results of EO tests, cyclic voltammograms of carbon paste electrode in *H. italicum* and *H. arenarium* extracts (methanolic extracts were diluted with phosphate buffer pH 7.3 before measurements) showed the presence of anodic currents at potentials above 0.1 V (Figure 1). However, the presence of electroactive substances with E_pa1_ at about 0.28 V and E_pa2_ at about 0.47 V were more clearly observed in the case of extract of *H. italicum* inflorescences (Figure 1, dash–dot line). In the reverse scan, an increase of cathodic current below 0.35 V was recorded, indicating that certain oxidized compounds were reduced.

**Figure 2:**
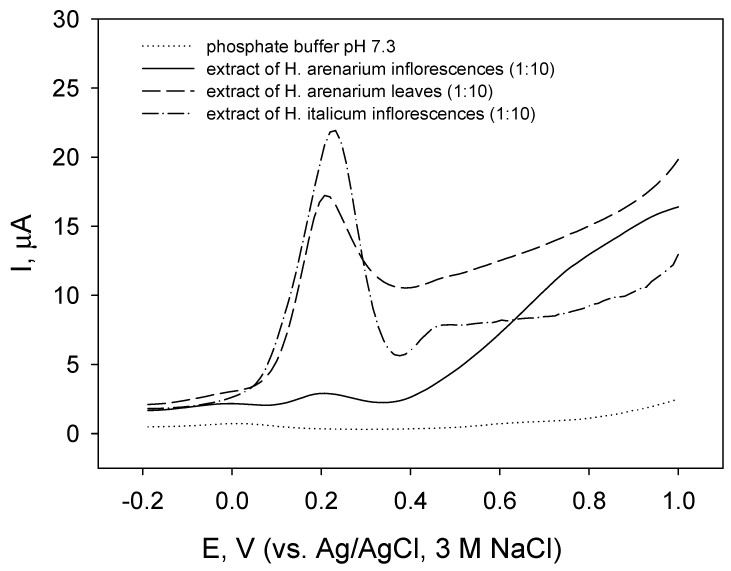
Square wave voltammograms of carbon paste electrode in extracts of inflorescences and leaves of *H. arenarium* and inflorescences of *H. italicum* (as indicated) in phosphate buffer pH 7.3; potential step 4 mV, amplitude 50 mV, frequency 25 Hz.

The voltammetric profiles in square wave voltammograms (Figure 2) revealed I_pa1_ of different magnitudes at E_pa1_ at 0.23 V for all extracts, whereas I_pa2_ at E_pa2_ at 0.46 V was observed only for extract of inflorescences of *H. italicum*.

### 2.5. Toxic Activity

A toxicity test employing brine shrimp *Artemia salina* (L.) (Anostraca: Artemiidae) larvae and *H. italicum* and *H. arenarium* EOs revealed that LC_50_ and LC_95_ values were in the range of 15.99–23.42 and 43.73–83.82 µg/mL, respectively (Table 5). Italian immortelle inflorescence and sandy everlasting leaf and flower EOs were found to be toxic, and were not statistically differentiated (*p* > 0.05).

Toxicity tests of methanolic extracts (up to 300 µL) of both *Helichrysum* species performed by the same method showed that the extracts were non-toxic.

## 3. Discussion

Among 53 identified constituents, aliphatic acids—palmitic (hexadecanoic, 23.8 ± 1.13%), myristic (tetradecanoic, 14.9 ± 1.05%), and lauric (dodecanoic, 6.1 ± 1.35%)—were determined as the major constituents in *H. arenarium* inflorescence oil (Table 1). In terpenoid class, trans-β-caryophyllene (5.4 ± 0.55%) and phytone (4.4 ± 0.55%) were the most abundant compounds. Sandy everlasting leaf EO contained comparable amounts of palmitic acid (18.8 ± 0.70%), myristic acids (8.7 ± 1.35%) and trans-β-caryophyllene (6.5 ± 0.55%), but differed in quantities of α-pinene (4.2 ± 1.15%) and *n*-nonanal (10.4 ± 1.50%) (Table 1). High content of fatty acids was obtained by the conventional hydrodistillation method (for 2 h). In order to extract these acids, a derivatization procedure is required in some cases [89].

It should be mentioned that the number of investigations on sandy everlasting EOs from Lithuania is limited [80,81,82], and the results obtained in this study differed from those of previously analysed EOs. Investigation of compositional variability of EOs from yellow and orange inflorescences (f. *aurantiacum* (Pers.) Bleck.) and leaves, collected from two limited wild populations in Eastern Lithuania, revealed principal constituents such as: aliphatic hydrocarbons heneicosane (≤32.1%) and octadecane (8.9%), sesquiterpenes β-caryophyllene (≤25.6%), and δ-cadinene (9.7 and 14.4%) [80]. The oils isolated from inflorescences of different six colors (citric, citric-yellow, yellow, yellow-brown, orange, orange-brown) of 11 *H. arenarium* accessions, originating from wild populations in Lithuania and propagated by clone sprouts and grown in a field collection in the Botanical Garden (Vilnius), were found to be rather different [81]. β-Caryophyllene (≤8.8%), δ-cadinene (≤8.2%), and heneicosane (5.0%) were prevalent in the EOs of citric-yellow, orange, and brown-orange inflorescences. Aliphatic hydrocarbon nonadecane (12.2%) was the main constituent of oils from yellow-brown flowers. Tetradecanoic acid (7.8%) dominated in the EOs from yellow inflorescences; an appreciable amount (7.0%) of this aliphatic acid was found in the EO of yellow-brown flowering tops [81]. It was recently reported [82] that EOs of sandy everlastings collected in coniferous woods from seven localities in Lithuania were characterized by β-caryophyllene (≤36.2%), octadecane (≤22.3%), heneicosane (≤20.0%) δ-cadinene (≤9.0%), 1,8-cineole (≤8.9%), and γ-cadinene (≤5.8%).

A comparison of the results of chemical composition obtained in the present study with those from other countries is quite complicated because of the limited number of published papers on this topic (for more details, see Table S2 in Supplementary Materials). Some compositional similarities were observed for plant material of Lithuanian origin and for *H. arenarium* of Caucasian origin cultivated in Hungary (Soroksár) and the Polish commercial sample [78,79]. The latter inflorescence oils contained significant amounts of aliphatic acids (up to 34.6%). Methyl palmitate (28.5%), dodecanoic (11.9%), decanoic acid (9.8%), and octanoic acids (6.0%) have been determined as predominant compounds in the Hungarian commercial sample [78]. Prevalent amounts of methyl palmitate (≤28.5%), capric (≤19.8%), lauric (≤14.6%), pelargonic (≤6.9%), and caprylic acid (6.0%) have been determined in plants cultivated in Hungary [79]. Methyl pentadecanoate (31.0%) and oleic acid (30.3%) along with ethyl hexadecanoate (20.2%) and linoleic acid (18.9%) were determined as major constituents, respectively, in flower EO of *H. arenarium* plants cultivated in Italy and plants grown naturally in Turkey [83,84] (see Table S2, Supplementary Materials).

Moreover, our results (Table 1) differed drastically from those obtained for sandy everlasting EOs of Serbian [56], Iranian [64], and Chinese origin [74], where monoterpene and sesquiterpene hydrocarbons were found to be the main components (details in Supplementary Materials, Table S2).

The data obtained clearly demonstrated remarkable differences in the chemical composition of the oils of Lithuanian *H.* *arenarium* L. from the sandy everlasting EOs of from other countries.

Italian immortelle inflorescence EO obtained from commercial herbs used in this study was characterized by principal compounds sesquiterpene hydrocarbons: γ-curcumene (21.5 ± 2.50%), β-selinene (13.6 ± 1.65%), β-eudesmol (8.3 ± 0.35%), α-selinene (8.1 ± 0.55%), and monoterpene α-pinene (6.5 ± 1.50%). Thirty-two identified compounds comprised 89.4 ± 0.15% of the total *H. italicum* EO (Table S3 in Supplementary Materials). The chemical composition of the investigated EO differed from *H. italicum* EOs of neryl acetate chemotype revealed in numerous studies (Table S1 in Supplementary Materials). On the other hand, γ- or *ar*-curcumene, α*-* or β-selinene were common constituents in Italian immortelle EOs.

Chemical composition of the EO obtained from a commercial inflorescence sample of Italian immortelle (*H. italicum*) differed drastically from EOs of Lithuanian wild sandy everlastings (*H. arenarium*) as well. Aliphatic acids predominated in *H. arenarium* oils, while sesquiterpene hydrocarbons were a major fraction in the *H. italicum* inflorescence EO. It should be mentioned that appreciable amounts of ester bonded acids and volatile carboxylic acids (after derivatization procedure) have been identified in EOs of *H. italicum* of Croatian origin [89]. Differences in chemical composition have demonstrated clear intraspecific variation of the different *Helichrysum* (*H. italicum* and *H. arenarium*) species.

According to the available literature data, around 100 compounds from different classes, such as phenolic acids, flavonoids, phthalides, arzanol derivatives and other pyrones, coumarins, sterols, lignans, etc., have been identified in various sandy everlasting extracts [45,49,63]. Even more, some compounds have been isolated and identified for the first time in *H. arenarium* extracts [67,68,73,75,76,77]. Twenty-nine compounds were identified tentatively in *H. arenarium* flower and leaf extracts in the present research, and most of them have been already identified in *H. arenarium* extracts or in other *Helichrysum* species. Fifteen constituents (bitalin A, dihydrosyringin, chlorogenic acid, unknown 1, caffeic acid, two naringenin glucoside isomers, syringin, dicaffeoylquinic acid, luteolin glycoside, apigenin-7-*O*-gentiobioside or apigenin-7,4‘-di-*O*-β-glucoside, apigenin, arenol, arzanol, and isosalipurposide) provided *m*/*z* ions on both ionization types, while some constituents were detected only by positive or by negative ionization. Because of very detailed and numerous published data on *H. italicum* extracts, the chemical composition of commercial Italian immortelle extract was not investigated in the present study.

The DPPH^●^ and ABTS^●+^ assays are most frequently used to evaluate the ability of antioxidants to scavenge free radicals. As determined by these tests, the activity of EOs from both *Helichrysum* species ranged from 0.25 ± 0.00 to 0.46 ± 0.01 for TROLOX (mmol/L) equivalent (Table 3 and Table 4) and practically did not vary with species, plant organs, and year of raw material collection.

Significant difference was observed between sandy everlasting EOs and methanolic extracts, the activity of leaf extract being 3-fold higher compared to that of inflorescence extract (DPPH^●^ assay, Table 3). ABTS^●+^ assay results showed the same tendency: activity of sandy everlasting flowers and leaf EOs was similar as well, activity of methanolic leaf extracts was also significantly higher (about six-fold) compared to that of flowers (Table 4).

The low ability to scavenge free radicals suggested that EOs of different *Helichrysum* species or plant parts did not have significant amounts of specific compounds that could successfully scavenge free radicals. For comparison, radical scavenging activity of *Rhododendron tomentosum* EOs was 48.19 ± 0.1 mmol/L (TROLOX equivalent) [90].

Several papers have been devoted to the antioxidant activity of *H. italicum* EOs and various extracts [17,18,27,30,33,34,35,39,42,44,45]. Comparison with our results is difficult because of variation in preparations of samples, the assays applied, and different ways of expression of the activity. The main tendency is that EOs of Italian immortelle exhibit weak/moderate antioxidant activity [17,27,30,35,42], while different extracts generally possess a good or even significant radical scavenging activity [18,27,30,33,39,43,44,45]. Contrary results were reported by Gismondi et al. [34]. EO from *H. italicum* flower heads containing principal constituents neryl acetate (33.97%), α-pinene (28.50%), nerol (7.97%), neryl phenylacetate (7.11%), and β-caryophyllene (5.71%) had a strong antioxidant activity, 90-fold and 250-fold more active than *Valeriana* sp. and *Nigella* sp. EOs, respectively [34] and references therein. Overall, it was concluded that the above immortelle EO possessed the highest antioxidant properties among the EOs extracted from other specimens of *H. italicum* or *Helichrysum* species [34].

There are no available data on antioxidant activity of EOs from *H. arenarium.* Data on radical scavenging potential of extracts from sandy everlasting are limited [45,52,53,54,55,57,60,63]. *Helichrysi flos* water extracts exhibited antioxidant and antilipoperoxidant properties; the plant extracts diminished enzymatically induced lipid peroxidation and reduced NADPH cytochrome *c* reductase activity in liver microsomes [52]. The hydrogen-donating ability and the reducing power of the lyophilized water extracts from inflorescences of sandy everlasting were determined spectrophotometrically; their OH^●^ scavenging activity in the H_2_O_2_/OH^●^–luminol–microperoxidase system was measured by a chemiluminometric method [53]. One lyophilizate proved to be more effective in scavenging DPPH radicals compared with silibinin used as a standard [53]. Various extracts (methanolic, ethanolic and 70% *v*/*v*, ethanolic extracts, before and after acid hydrolysis) of Romanian sandy everlasting (collected from Botoșani county), containing high amounts of polyphenols were tested for antioxidant properties [63]. The highest activity (5.82 TROLOX equivalent/mL extract) was found for the 70% (*v*/*v*) ethanolic extract of the flowers of *H. arenarium* and the lowest in the absolute ethanol-mediated extraction samples (3.71 TROLOX equivalent/mL). Antioxidative properties of infusions from *H. italicum* and *H. arenarium* plants were compared [45]. *H. italicum* infusions exhibited stronger radical scavenging activity than that of *H. arenarium* plants; and it was dependent on the morphological type of the plant, on the harvesting time and plant organ. Radical scavenging activity measured by the DPPH^●^ test in infusions prepared from green parts of *H. arenarium* was stronger than that from flowers [45].

Although DPPH^●^ and ABTS^●+^ scavenging assays in vitro are very popular, critical evaluation of these assays [91] pointed to their main limitation, i.e., these compounds are not found in living organisms. Besides, the following other considerations argue against direct polyphenol reactions with radicals in vivo: low concentrations of polyphenols in tissues, high level of metabolism, and biotransformation that polyphenols undergo in the organism, slow action (minutes or hours) as a radical scavenger of an antioxidant must be irrelevant in vivo in cells or even in situ in foods and etc. As an alternative to radical-scavenging assays, quick, simple, and inexpensive electrochemical techniques such as cyclic voltammetry, differential pulse or square wave voltammetry have been employed for evaluation of antioxidant properties of beverages, plant extracts, or individual polyphenols [92,93,94,95,96]. Electrochemical approaches are based on the chemico–physical properties of the compounds and, thus, can be considered a direct test for antioxidant properties.

Similar voltammetric profiles (Figure 1 and Figure 2) indicate that the extracts could contain the same compounds. Substances with pH-dependent oxidation potentials falling in the potential region from 0.2 to 0.5 V at pH 7 are possibly compounds containing a flavonoid structure with catechol or galloyl groups [92,93,94,95,97,98]. According to the results presented in Table 2, probable candidates are chlorogenic acid, caffeic acid, kaempherol, luteolin, and apigenin. Direct comparison of obtained E_pa_ values with literature data is rather complicated since the conditions of the experiment (electrode material, ionic strength of the solution, the presence of organic solvent, concentration of electroactive substance, etc.) may cause peak shifts [97]. Higher values of I_pa_ in voltammograms for leaf extracts (Figure 1 and Figure 2, dashed lines) compared to those for inflorescence extracts (Figure 1 and Figure 2, solid lines) correlated with higher total polyphenolic content in leaf extract. In the case of EOs, the non-appearance of anodic currents suggested the absence of easily oxidizable compounds that correlated with weak radical scavenging properties of *H. arenarium* EOs.

In vivo toxicity tests were performed using brine shrimp (*Artemia salina)* larvae [99]. LC_50_ values varied from 15.99 to 23.42 µg/mL for *H. italicum* and *H. arenarium* EOs, respectively (Table 5). According to Meyer’s and Clarkson’s toxicity criterion, plant extracts with LC_50_ < 1000 μg/mL are considered as toxic and extracts with LC_50_ of 0–100 μg/mL are highly toxic, respectively [100]. Regarding the toxicity classification with Meyer’s and Gosselin, Smith and Hodge’s criteria, the investigated EOs of *H. italicum* and *H. arenarium* could be attributed to the toxic/moderately toxic class [100]. Although the chemical composition of the EOs of two *Helichrysum* species was different and LC_50,95_ values varied, their toxic activities appeared to be similar (*p* > 0.05) when data were analysed statistically. To the best of our knowledge, researches devoted to evaluation of in vivo toxic capacity of both *Helichrysum* species (*H. arenarium* and *H. italicum*) are scarce. *H. italicum* EO did not show a mutagenic effect against larvae of *Drosophila melanogaster* Meigen (Diptera: Drosophilidae); however, co-incubation of the larvae with EO and urethane showed significant reduction of mutations caused by urethane [101]. EOs from *H. italicum* possessed high larvicidal activity against the mosquito *Aedes albopictus* (Skuse) (Diptera: Culicidae) [9] and displayed contact toxicity against the stored food insect *Sitophilus zeamais* Motsch (Coleoptera: Curculionidae) [47]. Investigation of in vitro phytotoxic activity of EO from *H. italicum* ssp. *italicum* on germination of radish and garden cress [7] showed that EO was active (probably due to the presence of bioactive sesquiterpenes) against radicle elongation of radish. Antifungal and antibacterial activity of *H. arenarium* and *H. italicum* EOs was among the most investigated properties of these oils [15,21,22,24,27,32,33,35,36,37,38,40,43,56,57,58,61,62,63,64,66]. The activity ranged from weak to strong, depending on EO and fungi/bacteria species.

The toxic activity of *H. arenarium* and *H. italicum* EOs was investigated for the first time in this study.

In vivo toxic activity of different amounts of methanolic extracts (30, 50, 100, and 300 µL) of both *Helichrysum* species was investigated by the same *Artemia salina* larvae method [99]. Mortality of brine shrimp (*Artemia salina)* larvae was observed neither after 24 h nor after 48 h. It appeared that the extracts were not only non-toxic but, conversely, could possibly be used as a nutrient medium for shrimps.

## 4. Materials and Methods

### 4.1. Plant Material

*H. arenarium* plants (up to 0.7 kg) were collected at full flowering stage (in August 2020 and 2021) in the forest site near Sudeikiai village (Utena district, Eastern Lithuania, 55°35′00.9″N 25°40′36.3″E). Area of the investigated population was up to 70 m^2^. The habitat is depicted on the geographic information system map (Figure S1 in Supplementary Materials). Raw material was taken immediately to the laboratory and dried at room temperature (20–25 °C) and under shade conditions; leaves and inflorescences were separated before drying. Plant material was identified by Dr. M. Rasimavičius and voucher specimen was deposited at the Vilnius University Herbarium (WI), Lithuania with a code number P33610.

Commercial sample of dried *H. italicum* inflorescences was purchased from Farmalabor (Farmacisti associate, Canosa di Puglia, Italy) producer.

### 4.2. Essential Oil Isolation

The essential oils were isolated by hydro-distillation of dried material (50 g each) in a Clevenger-type apparatus for 2 h, as per the European Pharmacopoeia. The ratio of plant material to water was 1:20. A yellow-grey, greasy mass with a sweet characteristic odor was obtained. Hydrodistillation yielded 1.1% (*v*/*w*, on a dry weight basis) of EO from *H. italicum* inflorescences. Yield of the *H. arenarium* EOs slightly ranged and was less than 0.5% (*v*/*w*). The obtained oils were dried over anhydrous sodium sulphate, kept in closed dark vials, and stored in a refrigerator; the samples were diluted with a mixture of pentane and diethyl ether (1:1) before analysis.

### 4.3. Preparation of Extracts

Samples of air-dried inflorescences and leaves were ground into a homogenous powder and protected from light and humidity until analysis. Preparation of extract was made according to Pharmacopeia requirements. 2 g of crushed herbal material (flowers and leaves) and 40 mL of solvent (mixture of water and methanol (1:1)) were used for extraction. Extraction procedure was performed in an ultrasonic bath at room temperature for 30 min. The mixture was filtrated through a filter paper for qualitative analysis (pore size 11 µm).

### 4.4. GC (Flame-Ionization Detector FID) Analysis

Quantitative analyses of the essential oils were carried out on HP 5890II chromatograph equipped with an FID (Hewlett Packard, Palo Alto, CA, USA), using DB-5 ((5%-phenyl)-methylpolisiloxane; 50 m × 0.32 mm × 0.25 µm) and HP-FFAP (polyethylene glycol 30 m × 0.25 mm i.d., film thickness 0.25 µm) capillary columns (Agilent, J&W Scientific, Santa Clara, CA, USA). The GC oven’s temperature was programmed as follows: increased from 50 °C (isothermal for 1 min) to 160 °C (isothermal for 2 min) at a rate of 5 °C/min, then increased to 250 °C at a rate of 10 °C/min; the final temperature was kept for 4 min. The temperature of the injector and detector was maintained at 250 °C. The flow rate of the carrier gas (hydrogen) was 1 mL/min. At least 2 repetitions (*n* ≥ 2) per analysis were performed.

### 4.5. GC-MS Analysis

Analyses were performed on a chromatograph Shimadzu GC-2010 PLUS (Shimadzu, Kyoto, Japan) interfaced to a Shimadzu GC-MS-QP2010 ULTRA mass spectrometer (Shimadzu, Kyoto, Japan) and fitted with a capillary column Rxi-5MS (Restek, Bellefonte, PA, USA), (5%-phenyl)-methylpolisiloxane 33 m × 0.25 mm i.d., film thickness 0.25 µm). The conditions of chromatographic separation were the same as for the GC (FID) analysis. The temperature of the injector and detector was 250 °C. The flow rate of carrier gas (helium) was 1 mL/min, split 1:20. At least 2 repetitions (*n* ≥ 2) per analysis were performed. The temperature of ion source was 220 °C. Mass spectra in electron mode were generated at 70 eV, 0.97 scans/second, mass range 33–400 *m*/*z*.

### 4.6. Identification of Individual Components

The percentage composition of the essential oils was computed from GC peak areas without correction factors. Qualitative analysis was based on comparison of retention indexes on both columns (polar and non-polar), co-injection of some reference terpenoids (α-, β-pinene, 1,8-cineole, linalool, camphor, β-caryophyllene, α-humulene and caryophyllene oxide), and C8-C28 n-alkane series; and mass spectra with corresponding data in the literature [85] and computer mass spectra libraries (Flavour and Fragrance of Natural and Synthetic Compounds 2 (FFNSC 2), Wiley and NIST). Identification was approved when the computer match with mass spectral libraries was with probabilities above 90%. The relative proportions of the oil constituents were expressed as percentages obtained by peak area normalization, all relative-response factors being taken as one.

### 4.7. HPLC-DAD-MS (TOF) Analysis

Methanolic extracts from *H. arenarium* inflorescences and leaves were analyzed by HPLC technique using a system HPLC/Diode Array Detector (DAD)/Time of Flight (TOF) (Agilent 1260 Infinity (Agilent Technologies, Waldbronn, Germany) and Agilent 6224 TOF (Agilent Technologies, Santa Clara, CA, USA)) equipped with a reverse phase column ZORBAX Eclipse XDB (C18, 5 µm particle size, 150 × 4.6 mm, Agilent Technologies, Santa Clara, CA, USA). The column temperature was maintained at 35 °C. Gradient system was applied: A (deionized water, containing 0.1% of formic acid) and B (acetonitrile, containing 0.1% of formic acid). Chromatographic separation was performed at a flow rate of 0.5 mL/min in the HPLC system by the following stepwise gradient elution method: initial 95%(A)/5%(B); from 0 to 20 min from initial ration to 0% (A)/100%(B); from 20 to 25 min: isocratic mode at 0%(A)/100% (B), from 25 to 30 min: from 0%(A)/100% (B) to 95%(A)/5%(B); and from 30 to 35 min isocratic mode at 95%(A)/5% (B). Ionization was performed by electrospray ionization interface (ESI) in positive or negative mode. Sample volume of 4 µL was injected by an auto-sampler.

MS (TOF) acquisition parameters were as follows: mass range 100–1700 *m/z*, acquisition rate 1.42 spectra/s, time 704.2 ms/spectrum. Ionization source conditions were as follows: drying gas temperature 350 °C, drying gas flow rate 3 L/min, nebulizer 15 psig, fragmentor voltage 125 V, skimmer 65 V. To assure the mass accuracy of recorded data, continuous internal calibration with reference masses *m/z*: 121.050873, 149.02332, 322.048121, 922.009798, 1221.990637, and 1521.971475 (as per instrument standards, ref. nebulizer 5 psig) was performed.

DAD range was set from 190 to 600 nm with selected scans at 254, 280, 320, 365, 380, 430, 480, and 515 nm, using 360 nm as reference abundance.

### 4.8. Determination of Total Phenolic Content (TPC)

The total phenolic content of *H. arenarium* methanol/water (1:1) extracts (from leaves and inflorescences) was determined using Folin-Ciocalteu assay [102]. 20 μL of methanolic/water extract and 1580 μL of distilled water was added to 100 μL Folin Ciocalteu reagent and 300 μL of Na_2_CO_3_ (20% *w*/*v*). The mixture was left in the darkness at room temperature for 2 h. The absorbance at 765 nm wavelength was measured using a spectrophotometer (UV/Vis Lambda 25, Perkin Elmer, Buckinghamshire, UK). The results are expressed as mg/L gallic acid equivalent. Calibration curve used for calculations (Figure S2 in Supplementary Materials) was obtained using different concentrations of gallic acid 0.00; 50; 100; 150; 250 and 500 mg/L. All measurements were done in triplicate.

### 4.9. Antioxidant Activity

#### 4.9.1. Spectrophotometric Assays

##### Antioxidant Capacity ABTS^●+^ Assay

Antioxidant capacity ABTS^●+^ assay was applied for *H. arenarium* inflorescence and leaf EOs and methanolic extracts. The stock solution containing ABTS^●+^ (2,2’-azino-bis(3-ethylbenzotiazoline-6-sulfonic acid) diammonium salt) and potassium persulfate (K_2_S_2_O_8_) was prepared dissolving these materials in a mixture of methanol and water (80:20) and left in the darkness for 12 h [33]. The working solution was prepared by diluting stock solution with a mixture of methanol and water (80:20) to obtain an absorbance value of 0.730 ± 0.02 at 734 nm. The absorbance was measured using the spectrophotometer (UV/Vis Lambda 25, Perkin Elmer, Buckinghamshire, UK). Essential oils and extracts for analysis were diluted with a mixture of methanol and water (80:20); 0.1 mL of prepared sample was allowed to react with 3.9 mL of working ABTS^●+^ solution for 15 min in the darkness. Thereafter, the absorbance of the reacted mixture was measured. The results are expressed in mmol/L TROLOX equivalent. All measurements were done in triplicate.

##### DPPH^●^ Assay

DPPH^●^ assay was applied for *H. arenarium* (inflorescence and leaf) and *H. italicum* inflorescence EOs and *H. arenarium* inflorescence and leaf methanolic extracts. 6 × 10^−5^ M stock solution of DPPH^●^ was obtained by dissolving 2,2-diphenyl-1-picrylhydrazyl with methanol. The working solution was prepared by diluting stock solution with methanol to obtain an absorbance value of 0.730 ± 0.02 at 515 nm. Essential oils and extracts for analysis were diluted with a mixture of methanol and water (80:20); 0.1 mL of the prepared sample was allowed to react with 3.9 mL of working DPPH^●^ solution for 30 min in the darkness [33]. Thereafter, the absorbance of reacted mixture was measured. The results are expressed in mmol/L TROLOX equivalent. All measurements were done in triplicate.

##### TROLOX Equivalent ABTS^●+^ and DPPH^●^ Assays

5 mg of TROLOX ((±)-6-hydroxy-2,5,7,8-tetra-methylchromane-2-carboxylic acid) was dissolved in a mixture of methanol and water (70:30) and diluted to 100 mL. To obtain standard calibration curves the solutions of five concentrations (200, 100, 50, 25, and 12.5 mmol/L) were prepared from this solution. 0.1 mL of each TROLOX solution was allowed to react with 3.9 mL of working solution of ABTS^●+^ and DPPH^●^. Absorbance values were measured after 15 and 30 min at 734 and 515 nm, respectively. Linear calibration curves (Figure S3 in Supplementary Materials) were obtained, and their parameters were used for further calculations of antioxidant activity. All measurements were done in triplicate.

#### 4.9.2. Electrochemical (Cyclic and Square Wave Voltammetry) Analysis

Cyclic and square wave voltammetry analysis at carbon paste electrode was applied for *H. arenarium* (inflorescence and leaf) and *H. italicum* inflorescence methanolic extracts. For EOs of both *Helichrysum* species, modified carbon paste electrode was used in this method.

Amperometric measurements were performed with BAS-Epsilon Bioanalytical system (West Lafayette, IN, USA). A conventional three-electrode cell contained carbon paste or essential oil-modified carbon paste electrode as a working electrode, platinum as an auxiliary electrode, and Ag/AgCl, 3 N NaCl as a reference electrode.

Carbon paste electrode was prepared by thoroughly mixing 200 mg of graphite powder with 100 µL of paraffin oil. EO-modified carbon paste electrode was prepared by mixing 100 mg of graphite, 50 µL of EO, and 50 µL of paraffin oil. The paste was packed into the cavity of a homemade electrode consisting of a plastic tube (2.9 mm) and a copper wire serving as an electrode contact. The surface of the electrode was thereafter smoothened on a white paper.

Phosphate buffer prepared from 0.025 M KH_2_PO_4_ contained 0.1 M KCl. The pH value was adjusted with KOH.

Cyclic voltammograms were recorded in the potential region—0.2 to 1.0 V at a potential scan rate 100 mV/s. Square wave voltammograms were recorded under the following conditions: step potential 4 mV, amplitude 50 mV, frequency 25 Hz.

### 4.10. Toxicity Test

Toxicity, usually expressed by LC_50_ values, is the degree to which chemical compound or mixtures of chemicals can damage an organism, a tissue, or a cell. Toxic activity of *H.* *arenarium* (inflorescences and leaves, separately) and *H. italicum* (inflorescence) EOs was tested in vivo, using brine shrimp *Artemia salina* (larvae) [99]. The eggs of shrimps hatch within 48 h to provide larvae (nauplii) in sea water (31 g sea salt per litre of water) at 20–25 °C. Thereafter, different concentrations of sand everlasting and immortelle essential oils dissolved in dimethyl sulfoxide were added. Survivors were counted after 24 h. Lethality (LC_50_ and LC_95_) of nauplii was calculated (*n* ≥ 4, with 95% confidence interval). Curves showing dependence of brine shrimp (*Artemia salina*) larvae lethality (%) on Log of EO in saline water are presented in Figure S4 in Supplementary Materials. Control tests were performed both with salt water and salt water with added DMSO (5–50 µL).

In vivo toxic activity of different amounts of methanolic extracts (30, 50, 100 and 300 µL) of both *Helichrysum* species was investigated by the same *Artemia salina* larvae method [99]. Survivors were counted after 24 and 48 h. The control tests were performed both with salt water and salt water with methanol (30–300 µL).

### 4.11. Statistical Analysis

The collected data were subjected to a one-way analysis of variance (ANOVA); the results were expressed as mean values, range intervals, and standard deviation (SD) values, using XLSTAT (trial version, Addinsoft 2014, Paris, France).

## 5. Conclusions

Chemical composition of EOs obtained by hydrodistillation from inflorescences and leaves of *H. arenarium* wild plants growing in Eastern Lithuania was reported. EOs containing palmitic (≤23.8%), myristic (≤14.9%) and lauric (6.1%) acids, *n*-nonanal (≤10.4%), and trans-β-caryophyllene (≤6.5%) differed from previously investigated sandy everlastings of Lithuanian origin. The data obtained clearly demonstrated remarkable dissimilarities in the chemical composition of the oils of *H.* *arenarium* (L.) Moench of Lithuanian origin and essential oils of sandy everlastings from other countries.

EO from flowers (commercial material) of *H.* *italicum* was investigated for comparison. The main components of the latter EO were γ-curcumene (21.5%), β- and α-selinene (13.6 and 8.1%, respectively), β-eudesmol (8.3%), and α-pinene (6.5%). This composition differed drastically from all EOs of Lithuanian wild *H. arenarium*. Aliphatic acids predominated in *H. arenarium* oils, while sesquiterpene hydrocarbons were a major fraction in the *H. italicum* inflorescence EO. Differences in chemical composition have demonstrated clear intraspecific variation of the different *Helichrysum* (*H. italicum* and *H. arenarium*) species.

Composition of *H. arenarium* methanolic extracts containing main compounds luteolin-7-*O*-glucoside, naringenin and its glucoside, apigenin, chlorogenic acid, arenol, and arzanol did not differ remarkably from previously investigated sandy everlasting extracts in other countries.

Low ability to scavenge free radicals (DPPH^●^ and ABTS^●+^ assays) suggested that EOs of different *Helichrysum* species or plant parts did not have significant amounts of specific compounds that could successfully scavenge free radicals. Methanolic extracts of *H. arenarium* leaves and inflorescences exhibited the ability to scavenge radicals, the activity of leaf extract being higher (up by about six-fold) compared to that of inflorescences.

Radical scavenging activities of extracts correlated with total polyphenolic content. I_pa_ values in voltammograms, i.e., electrochemical data, correlated with total polyphenolic content in *H. arenarium* extracts as well. The extracts showed a high level of total phenolic content and antioxidant potential, which make the studied plant species a potential source of natural antioxidants.

In the case of EOs, the non-appearance of anodic currents indicated the absence of easily oxidizable compounds that correlated with weak radical scavenging properties of *H. arenarium* and *H. italicum* EOs.

In vivo toxic activity of EOs and methanolic extracts of both *Helichrysum* species by brine shrimp bioassay was evaluated for the first time. *H. italicum* inflorescence and *H. arenarium* leaf and flower EOs was found to be toxic/moderately toxic, but did not differentiate statistically. Methanolic extracts of both species were not toxic.

## Figures and Tables

**Table 1:** The main chemical composition (constituents ≥ 3.0%) of essential oils obtained from *H. arenarium* inflorescences and leaves and *H. italicum* inflorescences.

Compound ^a^	^b^ *RIn_Lit_*	^c^ *RIn_exp_*	^d^ *RIp_exp_*	*H. aren.* Infl.	*H. aren.* Leaves	*H. ital.* Infl.
α-Pinene *	932	938	1035	0.3 ± 0.25	4.2 ± 1.15	6.5 ± 1.50
Limonene	1029	1030	1196	tr.	3.0 ± 0.50	1.6 ± 0.60
1,8-Cineole *	1031	1032	1218		3.9 ± 0.60	
*n*-Nonanal	1101	1103	1398	0.7 ± 0.20	10.4 ± 1.50	
*n*-Decanal	1202	1202	1503	0.9 ± 0.20	2.1 ± 2.0	
Italicene	1406	1406				2.2 ± 0.75
trans-β-Caryophyllene *	1419	1418	1608	5.4 ± 0.55	6.5 ± 0.55	3.3 ± 0.30
γ-Curcumene	1483	1486				21.5 ± 2.50
β-Selinene	1490	1490	1732			13.6 ± 1.65
α-Selinene	1498	1499				8.1 ± 0.55
Lauric acid		1577	2520	6.1 ± 1.35	2.0 ± 1.55	3.0 ± 0.15
β-Eudesmol	1649	1650	2237			8.3 ± 0.35
*n*-Tetradecanol	1673	1672	1917	2.8 ± 0.35		
Eudesm-7-(11)-en-4-ol	1700	1703				4.4 ± 0.40
Myristic acid		1741	2713	14.9 ± 1.05	8.7 ± 1.35	0.5 ± 0
Phytone		1838	2113	4.4 ± 0.55	1.4 ± 0.85	
Pentadecylic acid		1855	2820	2.1 ± 1.20	1.9 ± 0.95	
Palmitic acid		1945	2911	23.8 ± 1.13	18.8 ± 0.70	0.2 ± 0.06
Methyl linolenate		2075	2590	5.3 ± 0.75	2.7 ± 1.65	
*n*-Docosane	2200	2200		3.8 ± 0.35	0.3 ± 0.10	0.3 ± 0.06
Average Total				96.4 ± 1.52	99.1 ± 0.44	89.4 ± 0.15

^a^ Constituents are listed in order of their elution from a non-polar DB-5 (which is identical to a Rxi-5MS) column, compounds are identified by their mass spectra and retention indices on both (polar HP-FFAP and nonpolar Rxi-5MS) columns. * Additional identification with reference compound; tr.-traces (<0.05%). ^b^ *RIn_Lit_*: Kovat’s indices for the nonpolar column DB-5 taken from the literature [85]. ^c^ *RIn_exp_*: Retention indices determined experimentally on the nonpolar column Rxi-5MS (which is identical to DB-5). ^d^ *RIp_exp_*: Retention indices determined experimentally on the polar column HP-FFAP.

**Table 2:** Tentative identification of compounds in *H. arenarium* inflorescence and leaf extracts analyzed by HPLC-DAD-TOF.

Identity	t_R,_ min	Compound Formula	Molar Mass	*m/z* ESI+ (Da)	*m/z* ESI− (Da)
Bitalin A ^a^	3.1	C_13_H_14_O_3_	218.25	219.028	214.943
Bitalin A12-glucoside ^b^	3.2	C_19_H_24_O_8_	380.4	381.084	
Luteolin ^b^	6.7	C_15_H_10_O_6_	286.24	289.093	
5,7-Dihydroxyphthalide ^b^	7.3	C_8_H_6_O_4_	166.13	167.033	
Kaempferol ^b^	7.4	C_15_H_10_O_6_	286.24	289.090	
Dihydrosyringin ^b^	8.2	C_17_H_26_O_9_	374.39	375.093	371.008
Triptophan ^b^	8.2	C_11_H_12_N_2_O_2_	204.23	205.090	
Caffeoylquinic (chlorogenic) acid ^b^	8.4	C_16_H_18_O_9_	354.31	355.104	352.963
Unknown ^b^	8.5			707.180	706.984
Everlastoside E ^b^	8.6	C_19_H_28_O_11_	432.4	433.135	
Caffeic acid ^b^	8.7	C_9_H_8_O_4_	180.16	181.049	180.933
Apigenin-7-glucoside ^b^	8.7	C_21_H_20_O_10_	432.38	433.130	
Dimeric dihydrochalcone glycoside isomer ^b^	9.6	C_42_H_44_O_20_	868		867.013
Naringenin ^b^	10.1	C_15_H_12_O_5_	272.26	273.076	
Naringenin glucoside isomer 1 ^b^	10.2	C_21_H_22_O_10_	434.39	435.129	432.972
Dimeric dihydrochalcone glycoside isomer ^b^	10.2	C_42_H_44_O_20_	868		867.100
Syringin ^b^	10.5	C_17_H_24_O_9_	272.37	273.076	273.076
Dicaffeoylquinic acid ^a^	10.6	C_25_H_24_O_12_	516.4	517.135	514.959
Luteolin glycoside ^b^	10.8	C_21_H_20_O_11_	448.37	449.109	446.950
Naringenin glucoside isomer 2 ^b^	11.5	C_21_H_22_O_10_	434.4	435.129	432.976
Apigenin-7-*O*-gentiobioside/Apigenin-7,4′-di-*O*-β-glucoside ^b^	12.3	C_27_H_30_O_15_	594.5	595.145	592.956
Apigenin ^b^	13.7	C_15_H_10_O_5_	270.24	271.060	268.944
Arenol ^a,b^	18.0	C_21_H_24_O_7_	388.41	389.160	387.015
Arzanol ^a,b^	18.9	C_22_H_26_O_7_	402.4	403.175	401.026
Oleonolic acid ^a^	21.4	C_30_H_48_O_3_	456.7		455.064
Heliarzanol ^b^	21.9	C_24_H_30_O_8_	446.5		443.064
Resveratrol ^a^	22.7	C_14_H_12_O_3_	228.25		226.88
Isosalipurposide ^b^	23.2	C_21_H_22_O_10_	434.4	435.255	433.099
β-Sitosterol ^b^	25.1	C_29_H_50_O	414.71		413.132
Quercetin 3-*O*-malonyl glucoside ^a^	31.3	C_24_H_22_O_15_	550.4	550.63	

^a^ Compounds identified in *H. arenarium* leaf extracts. ^b^ Compounds identified in *H. arenarium* inflorescence extracts.

**Table 3:** Antioxidant activity of *H. arenarium* inflorescence and leaf, and *H. italicum* inflorescence essential oils and extracts using DPPH^●^ assay.

Equivalent, mmol/L	*H. aren.* Infl. EO (2021)	*H. aren.* Infl. EO (2020)	*H. aren.*Leaf EO (2020)	*H. aren.* Infl. Extract (2021)	*H. aren.*Leaf Extract (2021)	*H. ital.* Infl. EO
TROLOX	0.27 ± 0.01	0.25 ± 0.01	0.25 ± 0.001	6.13 ± 0.04	19.13 ± 0.04	0.35 ± 0.03

**Table 4:** Antioxidant activity of *H. arenarium* essential oils and extracts using ABTS^●**+**^ assay.

Equivalent, mmol/L	*H. aren.* Infl. EO (2021)	*H. aren.* Infl. EO (2020)	*H. aren.* Leaf EO (2020)	*H. aren.* Infl. Extract (2021)	*H. aren.* Leaf Extact (2021)
TROLOX	0.46 ± 0.01	0.40 ± 0.001	0.42 ± 0.01	1.96 ± 0.01	11.18 ± 0.002

**Table 5:** Toxic activity of *H. arenarium* and *H. italicum* essential oils.

*Artemia salina* Nauplii Lethality	*H. arenarium* Inflorescence	*H. arenarium* Leaf	*H. italicum* Inflorescence
LC_50_, µg/mL	23.42	21.97	15.99
LC_95_, µg/mL	83.82	82.66	43.73

*p*-values are greater that the significance level α = 0.05.

## Data Availability

Not applicable.

## Supplementary Materials

The following are available online. Table S1. Principal chemical composition (compounds ≥ 5.0%) of *Helichrysum italicum* (Roth) G. Don essential oils investigated in various countries over the last 10 years *, Table S2. Summary of previously reported data on chemical composition and bioactivity of essential oils of *H. arenarium* (L.) Moench) plants grown in different countries worldwide *, Table S3. Chemical composition of essential oils obtained from *H. arenarium* inflorescences and leaves and *H. italicum* inflorescences, Figure S1. Geographical indication of *H. arenarium* (L.) Moench sampling site in Eastern Lithuania (Utena district), Figure S2. Gallic acid standard calibration curve, Figure S3. TROLOX standard calibration curves: (a) DPPH˙ assay; (b) ABTS˙+ assay, Figure S4. Dependence of brine shrimp (*Artemia salina*) larvae lethality (%) on Log of essential oil concentration in saline water.

## Sample Availability

Samples of plant extracts and essential oils are available from the authors.
