# Supplementary material for: Antioxidant and Toxic Activity of Helichrysum arenarium (L.) Moench and Helichrysum italicum (Roth) G. Don Essential Oils and Extracts
Source: Molecules. 2022 Feb 15;27(4):1311. doi: 10.3390/molecules27041311 (PMC8879542; doi:10.3390/molecules27041311)
Supplement: Supplementary file 1 [file molecules-27-01311-s001.zip › molecules-1572766-SI.pdf]

molecules

MDPI

Supplementary Materials

Antioxidant and Toxic Activity of *Helichrysum arenarium* (L.) Moench and *Helichrysum italicum* (Roth) G. Don Essential Oils and Extracts

Asta Judzentiene [1,*], Jurga Budiene [1], Irena Nedveckyte [2] and Rasa Garjonyte [1]

[1] Department of Organic Chemistry, Center for Physical Sciences and Technology, Sauletekio Avenue 3, LT-10257 Vilnius, Lithuania; jurga.budiene@ftmc.lt (J.B.); rasa.garjonyte@ftmc.lt (R.G.)
[2] Institute of Biosciences, Life Sciences Center, Vilnius University, Sauletekio Avenue 7, LT-10257 Vilnius, Lithuania; irena.nedveckyte@gf.vu.lt.
* Correspondence: asta.judzentiene@ftmc.lt; Tel.: +370-69412190

Table S1. Principal chemical composition (compounds ≥5.0%) of *Helichrysum italicum* (Roth) G. Don essential oils investigated in various countries for last ten years *.

Plant Material	Main Constituents, %
20 native *H. italicum* ssp. *italicum* genotypes collected from different locations in Italy and Corsica (France) and grown under similar condition [5]	Several chemotypes were described. Neryl acetate (≤32.0), β- or α-selinene (≤38.0 and ≤26.5%, respectively), β-bisabolene (32.3), γ-curcumene (≤27.7), nerol (18.8), β-caryophyllene(≤18.6), caryophyllene oxide (14.9), carvacrol (≤14.8), β- or γ-eudesmol (13.7 and ≤20.4, respectively), *ar*-curcumene (≤8.3), neryl propanoate (6.8); 4,6, 9-trimethyldec-8-en-3, 5-dione (≤11.0); 5, 7, 10-trimethylundec-9-en-4,6-dione (≤6.9)
ssp. *microphyllum* from Sardinia, under different weather conditions, from their vegetative period to post-blooming time [6]	Neryl acetate (≤35.6), eudesmen-5-en-11-ol (≤23.5), nerol (≤14.4)
ssp. not indicated, from Elba Island [7,9]	Neryl acetate (25.3), α-pinene (14.5), limonene (12.3), γ-curcumene (8.7),
ssp. not indicated, flowers from Konavle region, Croatia [8]	γ-Curcumene (12.4), β-selinene (9.9), α-selinene (5.9)
ssp. *italicum* from the National Park of Cilento and Diano Valley, Southern Italy [10]	*iso*-Italicene epoxide (16.8), β-costol (7.5)
ssp. *italicum* from Corsica, France [11]	Neryl acetate (31.0), γ-curcumene (10.7), neryl propionate (5.1)
ssp. not indicated, collected in 7 locations of Elba Island (Tuscany, Italy) [12]	Neryl acetate (≤45.9), nerol (≤12.8)
Diversity of 50 *H. italicum* (ssp. not indicated) populations collected in Sardinia [13]	Neryl acetate, nerol, neryl propionate, eudesm-5-en-11-ol, γ-cadinene, *cis*-β-guaiene, nerolidol
Flowers (ssp. not indicated) collected in the region Konavle, Southern part of Croatia [14]	CO_2 extracts: octacosane (10.2), canellal (10.0), cembrenol (7.4), santonine (6.6)<br>EO: α-pinene (5.0)
Commercial EO sample of *H. italicum* (ssp. not indicated) from Herba d.o.o, Belgrade, Serbia [15]	γ-Curcumene (22.5), α-pinene (15.9), neryl acetate (7.8), β-selinene (6.9)
ssp. not indicated, collected from 4 populations (on Brač Island, Biokovo Mt. near Tijarica and near Makarska) in Dalmatia, Croatia [16]	α-*trans*-Bergamotene (10.2), β-acoradiene (10.1), rosifoliol (8.5), neryl acetate (8.1)
ssp. *italicum* collected in Montenegro [17]	Neryl acetate (28.2), γ-curcumene (18.8), neryl propionate (9.1), *ar*-curcumene (8.3)

ssp. *picardii* collected from field-growing plants at Vila Real de Santo António, Algarve, Portugal [18]	α-Pinene (53.5), γ-curcumene (27.4)
ssp. *italicum*,<br>8 EOs from Sicily, plants harvested in the oriental region of Etna volcano; 2 EOs from southern Corsica [19]	Sicilian EOs: β-selinene (14.0), rosifoliol (13.9), α-selinene (11.1), aromadendrene (11.0), ledol (8.6), β-caryophyllene (7.6), α-pinene (7.1)<br>Corsican EOs: neryl acetate (46.2), α-pinene (12.5), γ-curcumene (11.7)
146 *H. italicum* ssp. *microphyllum* genotypes from two contrasting habitats (seaside and mountains) in Sardinia [20]	Neryl acetate (≤83.4), *cis*-β-guaiene (58.2), (*E*)-nerolidol (≤55.6), eudesmen-7-(11)-en-4-ol (≤46.1), limonene (≤15.7), neryl propionate (≤14.1), nerol (≤13.6), γ-curcumene (≤10.6), 1,8-cineole (8.9), neryl-isobutanoate (7.2), *epi*-cadinol (6.7), δ-cadinene (6.3)
var. *numidicum* Pomel from Bibans, Algeria [21]	Isopropyl tetradecanoate (12.1), α-pinene (12.0), hexadecanoic acid (10.0), (*E*)-caryophyllene (9.2), ledol (9.1), palustrol (5.6)
ssp. not indicated from Béjaia, North of Algeria [22]	α-Cedrene (13.6), α-curcumene (11.4), geranyl acetate (10.1), limonene (6.1), nerol (5.0)
ssp. not indicated, commercial EO sample from doTERRA Intl., Pleasant Grove, UT, USA [23]	Neryl acetate (35.5), γ-curcumene (13.9), α-pinene (8.9)
ssp. not indicated, collected at continental part of Zadar and Šibenik-Knin County, Central Dalmatia, Croatia [24]	α-Pinene (21.6), γ-curcumene (21.6), neryl acetate (7.9), β-selinene (6.5), *iso*-italicene (5.4)
ssp. not indicated, plants (of Corsican orifgin) cultivated in Turia village, municipality of Pavel Banya, Stara Zagora region, South Bulgaria harvested in different phenological stages [25]	Citronellol isobutanoate (≤25.8), neryl acetate (≤20.6), α-pinene (≤19.5), α-copaene (≤12.8), eudesm-5-en-11-ol (8.1), β-eudesmene (≤5.6)
ssp. *italicum*, commercial plant samples from Roing, Ljubuški, Bosnia and Herzegovina [26]	α-Pinene (≤29.4), limonene (≤5.5), neryl acetate (≤7.8), italicene (≤7.1), 2,4,6,9-tetramethyldec-8-en-3,5-dione B (7.1), β-selinene (≤5.6), α-curcumene (5.0)
ssp. not indicated, collected near Benkovac, Croatia [27]	Neryl acetate (20.4), γ-curcumene (14.1), *trans*-α-bergamotene (7.0)
ssp. *italicum* cultivated in Marche region, Central Italy [28]	Neryl acetate (15.7), α-pinene (8.2), 4,6,9-trimethyl-8-decene-3,5-dione (italidione I) (7.3), *ar*-curcumene (5.4), β-selinene (5.4)
ssp. *italicum*, commercial EO from Donna è Company, Monticellu;<br>EO was produced from wild plants collected in the area of Lumiu, Northern Corsica [29]	Neryl acetate (30.4), γ-curcumene (10.1), neryl propionate (7.1), *ar*-curcumene (5.8)
ssp. *italicum* collected during five different periods in Herzegovina [30]	α-Pinene (15.7), γ-curcumene (12.8), 4,6,9-trimethyldec-8-en-3,5-dione (8.7), neryl acetate (6.9), limonene (6.4), β-selinene (5.3)
ssp. *italicum, c*ommercial EO from Casa Vecchia Corsa, Sartène, Corsica, France [31]	Neryl acetate (35.0), neryl propionate (5.6) in ester-containing chromatographic fraction
Commercial EO sample from Agro Life Montenegro d.o.o.<br>ssp. not indicated, plants collected in Tuzi near Podgorica, Montenegro [32]	In liquid EO: β-eudesmene (21.6), β-bisabolene (19.9), α-pinene (16.9), neryl acetate (10.7)<br>In the vapour EO phase: α-pinene (78.8)
two subspecies (ssp. *microphyllum* (plants origin from Bosnia) and ssp. *italicum* (French origin)) cultivated in the area of Kazanlak, Bulgaria [33]	ssp. *microphyllum*: α-pinene (20.8), γ-curcumene (16.5), β-selinene (5.6)<br>ssp. *italicum:* neryl acetate (33.9), γ-curcumene (8.8), rosifoliol (5.5), geranyl propionate (5.0),

ssp. not indicated, from Botanical Garden of the University of Rome, Italy [34]	Neryl acetate (34.0), α-pinene (28.5), nerol (8.0), neryl phenylacetate (7.1), β-caryophyllene (5.7)
ssp. not indicated, flowering parts of *H. italicum* introduced from Ćapljina (Bosnia and Hercegovina) and cultivate in the experimental fields of the Institute of Field and Vegetable Crops (IFVCNS) in Bački Petrovac, Novi Sad, Serbia [35]	γ-Curcumene (13.6), β-selinene (12.2) α-pinene (11.8), β-caryophyllene (6.7), neryl acetate (5.5)

*The published data presented by chronological order (from 2010 to 2021).

Table S2. Summary of previously reported data on chemical composition and bioactivity of essential oils of *H. arenarium* (L.) Moench) plants grown in different countries worldwide *.

Plant Material	EO Preparation Procedure	Main constituents (≥5.0), %	Biological Activity, Evaluation Method, Results
Inflorescences of *H. arenarium* (Helichrysi flos - GYNKI B 145/98 MSZ 1.0.) collected in the region of Caucasus were purchased from Hungarian commercial herb distributor [78]	EO was obtained by HD procedure described in the VII. Hungarian Pharmacopoeia. Yield of EO was 0.09%.	Methyl palmitate (28.5), dodecanoic acid (11.9), decanoic acid (9.8), octanoic acid (6.0)	Not investigated
Hungarian and Polish mercantile samples and a plant sample cultivated in Hungary (Soroksár) [79]	EOs prepared by steam-distillation	Methyl palmitate (8.0-28.5), capric acid (8.5-19.8%), lauric acid (6.5-14.6), δ-cadinene (5.2-7.9), thymol (7.7), pelargonic acid (5.2-6.9), carvacrol (6.2), caprylic acid (6.0)	Not investigated
Plants were collected from Kladovo, Serbia and Montenegro in the flowering period [56]	EO was obtained from the Faculty of Technology and Metallurgy, University of Belgrade (from S. Šiler-Marinković), other details not specified.	Di-epi-α-cedrene (17.9), α-ylangene (14.0), cyclosativene (11.9), limonene (11.4), α-selinene (8.2)	Antifungal activity by microdilution test using *Aspergillus niger*, *A. flavus*, *Cladosporium cladosporioides*, *Penicillium funiculosum* and *Trichoderma viride*. MIC = 10–30 μg/mL; MFC = 15–60 μg/mL. Antimicrobial activity against the Gram (+) (*Micrococcus luteus*, *Staphylococcus aureus*, *Staphylococcus epidermidis*) and Gram (−) bacteria (*Escherichia coli*, *Pseudomonas tolaasii*, *Salmonella enteritidis*, *Salmonella typhimurium*) and yeast *Candida albicans* by a bioautographic test on TLC plates. EO showed a strong antimicrobal activity: a

			volume of 1 μL had antimicrobial activity against all bacterial and yeast species tested.
H. arenarium plants with yellow and orange [f. *aurantiacum* (Pers.) Bleck.] flowers collected from the same natural habitats in Svencionys and Ukmerge districts, Eastern Lithuania [80]	HD for 2 h, using a mixture of hexane and diethyl ether as a collecting solvent.	Heneicosane (32.1), β-caryophyllene (13.5–25.6), δ-cadinene (9.7 and 14.4), octadecane (8.9)	Not investigated
Plants collected from natural populations, and cultivated in Vilnius, Lithuania. Aerial parts gathered at flowering stage from the field collection [81]	EO from inflorescences of six different colours: citric, citric-yellow, yellow, yellow-brown, orange, and orange-brown. HD for 3 h, a mixture of pentane and diethyl ether (1:1) used as a solvent.	*n*-Nonadecane (12.2), trans-caryophyllene (5.3–8.8), δ-cadinene (5.1–8.2), tetradecanoic acid (7.0 and 7.8), caryophyllene oxide (5.7), heneicosane (4.9–5.1)	Not investigated
Aerial parts in Khorasan Razavi Province (the Northeast of Iran) [62]	Essential oil of aerial parts of the *H. arenarium* L. was extracted by steam distillation using a Clevenger apparatus according to the method of British Pharmacopoeia.	Not detailed	*In vitro* on the growth of seven microbial species: *Bacillus subtilis, Escherichia coli, Staphylococcus aureus, Saccharomyces cerevisiae, Candida albicans, Aspergillus flavus* and *Aspergillus parasiticus,* using a micro-dilution method. Results showed that *B. subtilis* (MIC = 781.25 and MBC = 6250 μg/ml) was more resistance than the two other bacterial species. Among the two yeasts tested, *S. cerevisiae* (MIC = 97.65 and MFC = 781.25μg/ml) was more sensitive than *C. albicans* (MIC = 195.31 and MFC = 3125 μg/ml) while among the fungal species, growth of *A. parasiticus* inhibited at lower concentration of the EO rather than *A. flavus.* In liquid medium (Mullerhinton broth), EO exhibited the same MIC value against both fungal strains (48.82 μg/ml), while in solid medium (Mullerhinton

			agar) different activity was observed against *A. flavus* and *A. parasiticus* (MFC = 6250 and 390.625 µg/ml, respectively).
Plants collected in Khorasan Razavi Province, Northeast of Iran [64]	EO from aerial parts extracted by water steam distillation, using a Clevenger apparatus according to the method of British Pharmacopoeia. Other detailes not specified.	α-Pinene (32), 1,8-cineole (16), α-humulene (15), β-caryophyllene (8)	Antimicrobial activity *in vitro* on the growth of three food born pathogens: *Serratia marcescens*, *Streptococcus agalactiae* and *Staphylococcus aureus,* using a micro-dilution method. MIC: 406, 812 and 812 µg/mL for *S. marcescens*, *S. agalactiae* and *S. aureus;* respectively. MBC: 812, 3250 and 3250 µg/mL, respectively.
H. arenarium, native from different geographical areas, but acclimatised and grown in homogeneous conditions in Italy (CREA-Sanremo collection) [83]	EO extraction: leaves and flowers, separately from the three *Helichrysum* species were subjected to HD by Clevenger-type apparatus (3h). EOs had very low yield (≤0.01%), so they were collected with n-hexane (HPLC grade) and stored at −4 °C in the dark until use. Solid Phase Micro Extraction analyses (SPME): The analyses were performed using Supelco SPME device coated with polydimethylsiloxane (PDMS, 100 µm) for VOC emission of flowers and leaves.	In flower EO: methyl ester of pentadecanoic acid (31.0), β-caryophyllene (27.5), β-pinene (7.4) In leaf Eo: β-caryophyllene (46.0), 1,8-cineole (8.3), limonene (5.5) Volatile emission by SPME analysis: In flowers: β-pinene (55.2), α-pinene (15.8), limonene (5.2), 1,8-cineole (5.1); in leaves, (*Z*)-3-hexenol acetate (49.6), butanoic acid, (*Z*)-3-methyl-3-hexenyl ester (11.1), 1,8-cineole (7.4), β-pinene (5.8)	Not investigated
Plants collected at full flowering from 7 locations (Birstonas, Varena Alytus, Ukmerge, and Moletai districts, and Neringa city) in Lithuania, mainly in the coniferous woods [82]	Leaves and inflorescences of various intensities of the yellow colour. HD for 3 h, mixture of pentane and diethyl ether (1:1) used as a collecting solvent. The yield of EOs was ≤0.5%.	β-Caryophyllene (5.8–36.2), octadecane (7.1–22.3), heneicosane (7.9–20.0) δ-cadinene (9.0), 1,8-cineole (8.9), γ-cadinene (5.8)	Not investigated
The aerial parts were hand-harvested during flowering from Alax in Inner Mongolia, China [74]	By HD concatenated with a liquid-liquid extraction. Traditional Clevenger apparatus. was modified: two separation columns were	β-Spatulenol (19.9–24.0), ledol (6.2–10.0), bicyclogermacrene (5.7), aromadendrene (5.2)	Not investigated

	added. The first one is the conventional oil-water separation column, and the second one is the extraction column for EO components from hydrosol using organic solvents. The theoretical yield of the total EO was 5.10 mg/g.		
The aerial parts were collected in full flowering stage from Yozgat Akdagmadeni district (at altitude of 1760 m), Turkey [84]	HD for 2 h in a laboratory glass apparatus of the British Pharmacopoeia. The yield of EO was 0.07%.	Oleic acid (30.3), ethyl hexadecanoate (20.2), linoleic acid (18.9)	Not investigated

*The published data presented by chronological order (2000–2020); HD-hydrodistilation procedure.

Table S3. Chemical composition of essential oils obtained from *H. arenarium* inflorescences and leaves and *H. italicum* inflorescences.

**Compound** [a]	[b] RIn_{Lit}	[c] RIn_{exp}	[d] RIp_{exp}	***H. aren.* Flowers**	***H. aren.* Leaves**	***H. ital.* Flowers**
2-Heptanone	892	891		0.6 ± 0.25	0.1 ± 0	
n-Nonane	900	900		0.4 ± 0.10	0.3 ± 0.15	
Heptanal	902	903		0.3 ± 0.10	1.1 ± 0.40	
α-Pinene *	932	938	1035	0.3 ± 0.25	4.2 ± 1.15	6.5 ± 1.50
Camphene	954	955	1070		0.4 ± 0.30	
2-*Z*-Heptenal		959	1319		0.7 ± 0.20	
Benzaldehyde	960	964		1.6 ± 0.56		
β-Pinene*	979	978	1113	0.1 ± 0.21	0.8 ± 0.25	
1-Octen-3-ol	979	980			0.8 ± 0.10	
1-Octen-3-one		982	1285	0.1 ± 0.10		
2-Pentilfuran		996	1237	1.7 ± 0.55	2.3 ± 0.30	
trans-2-(2-Pentenyl)furan		1003	1284	0.4 ± 0.20	1.2 ± 0.25	
n-Octanal	999	1004		0.3 ± 0.30	0.9 ± 0.35	
Limonene	1029	1030	1196	tr.	3.0 ± 0.50	1.6 ± 0.60
1,8-Cineole*	1031	1032	1218		3.9 ± 0.60	
γ-Terpinene	1060	1060	1255	tr.	0.7 ± 0.70	
Linalool*	1097	1098	1550			2.1 ± 0.45
α-Campholenal	1126	1125			0.1 ± 0.06	
Camphor*	1146	1145	1518			0.5 ± 0.20
n-Nonanal	1101	1103	1398	0.7 ± 0.20	**10.4** ± 1.50	
2*E*-Nonen-1-al	1161	1160		0.5 ± 0.50	0.9 ± 0.25	
Isoborneol	1162	1164	1660	0.1 ± 0.10		0.4 ± 0.20
Terpinen-4-ol	1177	1181	1604		0.4 ± 0.25	
Octanoic (Caprylic) acid		1185	2070?	0.7 ± 0.65	0.2 ± 0.2	
α-Terpineol	1189	1188	1698	0.6 ± 0.45	1.3 ± 0.35	0.8 ± 0.40
n-Dodecane	1200	1200		0.2 ± 0.20		
Decanal	1202	1202	1503	0.9 ± 0.20	2.1 ± 2.0	
Geraniol	1253	1255	1845			1.3 ± 0.3
2*E*-Decenal	1264	1264			0.4 ± 0.40	
Nonanoic (Pelargonic) acid	1271	1269		0.8 ± 0.70	1.2 ± 0.25	

E-Anethole	1285	1285				1.1 ± 0.35
2-Undecanone	1294	1292		0.2 ± 0.15		
Undecanal	1307	1305	1602	0.4 ± 0.15	0.4 ± 0.35	
2*E*,2*E*-decadienal	1317	1315			1.5 ± 1.45	
Eugenol	1359	1363	2179	0.8 ± 0.25		
α-Ylangene	1375	1372	1491	0.2 ± 0.15	0.4 ± 0.15	
α-Copaene	1377	1376	1497	1.4 ± 0.60	2.1 ± 0.45	
n-Decanoic (Capric) acid		1382	2275	1.2 ± 0.55	1.1 ± 0.15	
E-β-Demascenone	1385	1388		0.5 ± 0.00		
n-Tetradecane	1400	1400		1.5 ± 0.25		
iso-Italicene	1402	1405				0.7 ± 0.15
Italicene	1406	1406				2.2 ± 0.75
n-Dodecanal	1409	1408		0.5 ± 0.35		
cis-α-Bergamotene	1413	1412				0.2 ± 0.06
E-Caryophyllene*	1419	1418	1608	5.4 ± 0.55	6.5 ± 0.55	3.3 ± 0.30
trans-α-Bergamotene	1435	1439				0.7 ± 0.06
Aromadendrene	1441	1440	1615	0.2 ± 0.1	0.2 ± 0.15	0.8 ± 0.30
α-Humulene*	1455	1455	1680	0.9 ± 0.2	0.4 ± 0.15	
Z-β-Farnesene			1645		1.8 ± 0.75	
α-Acoradiene	1466	1468	1670			0.5 ± 0.0
γ-Muurolene	1480	1478	1690	0.4 ± 0.31	1.1 ± 0.10	
γ-Curcumene	1483	1486				**21.5** ± 2.50
E-β-Ionone	1489	1501	1955		1.7 ± 0.75	
β-Selinene	1490	1490	1732			**13.6** ± 1.65
α-Selinene	1498	1499				**8.1** ± 0.55
n-Pentadecane	1500	1500		0.4 ± 0.15		
α-Muurolene	1500	1502		0.4 ± 0.35	0.6 ± 0.55	
γ-Cadinene	1514	1515	1764	0.6 ± 0.35	0.9 ± 0.90	0.8 ± 0.20
δ-Cadinene	1523	1520	1760	1.6 ± 0.35		1.3 ± 0.65
Italicene ether	1538	1533				0.6 ± 0.06
α-Cadinene	1539	1536	1775		0.3 ± 0.25	
α-Calacorene	1546	1545	1934	0.2 ± 0.15		
β-Calacorene	1566	1568			0.3 ± 0.30	
E-Nerolidol	1563	1567	2045		0.2 ± 0.20	
Caryophyllenyl alcohol	1572	1572			2.3 ± 0.35	
Dodecanoic (Lauric) acid		1577	2520	6.1 ± 1.35	2.0 ± 1.55	3.0 ± 0.15
Spathulenol	1578	1580				1.1 ± 0.10
β-Caryophyllene oxide*	1583	1584	2000	0.6 ± 0.35	1.2 ± 0.35	1.0 ± 0.15
Guaiol	1601	1602				1.1 ± 0.45
Humulene epoxide II	1608	1608	2053		0.2 ± 0.20	
1,10-di-*epi*-Cubenol	1619	1620			0.6 ± 0.60	
14-hydroxy-9-*epi*-*E*-Caryophyllene	1670	1670		0.4 ± 0.2	0.3 ± 0.30	
β-Eudesmol	1649	1650	2237			**8.3** ± 0.35
α-Cadinol	1654	1655	2249	0.7 ± 0.3	0.4 ± 0.35	
Selin-11-en-4-α-ol	1660	1660				0.7 ± 0.01
Tridecanoic (Tridecylic) acid		1671	2605			
n-Tetradecanol	1673	1672	1917	2.8 ± 0.35		
Eudesm-7-(11)-en-4-ol	1700	1703				4.4 ± 0.40
n-Tetradecanoic (Myristic) acid		1741	2713	**14.9** ± 1.05	**8.7** ± 1.35	0.5 ± 0
Phytone (Hexahydrofarnesyl acetone)		1838	2113	4.4 ± 0.55	1.4 ± 0.85	
n-Pentadecanoic (Pentadecylic) acid		1855	2820	2.1 ± 1.20	1.9 ± 0.95	

n-Hexadecanoic (Palmitic) acid		1945	2911	**23.8** ± 1.13	**18.8** ± 0.70	0.2 ± 0.06
Phytol	1943	1948		0.4 ± 0.10	0.2 ± 0.15	
n-Eicosane	2000	2000		0.3 ± 0.15	0.4 ± 0.10	
Methyl linolenate		2075	2590	5.3 ± 0.75	2.7 ± 1.65	
n-Heneicosane	2100	2100		2.1 ± 0.25	0.9 ± 0.25	0.2 ± 0.06
n-Docosane	2200	2200		3.8 ± 0.35	0.3 ± 0.10	0.3 ± 0.06
n-Tricosane	2300	2300		0.3 ± 0.12		
Average Total				96.4 ± 1.52	99.1 ± 0.44	89.4 ± 0.15

[a] Constituents are listed in order of their elution from a non-polar DB-5 (which is identical to a Rxi-5MS) column, compounds are identified by their mass spectra and retention indices on both (polar HP-FFAP and nonpolar Rxi-5MS) columns; * Additional identification with reference compound; tr.-traces (<0.05%). [b] RIn_{Lit}: Kovat's indices for the nonpolar column DB-5 taken from the literature [85]. [c] RIn_{exp}: Retention indices determined experimentally on the nonpolar column Rxi-5MS (which is identical to DB-5). [d] RIp_{exp}: Retention indices determined experimentally on the polar column HP-FFAP.

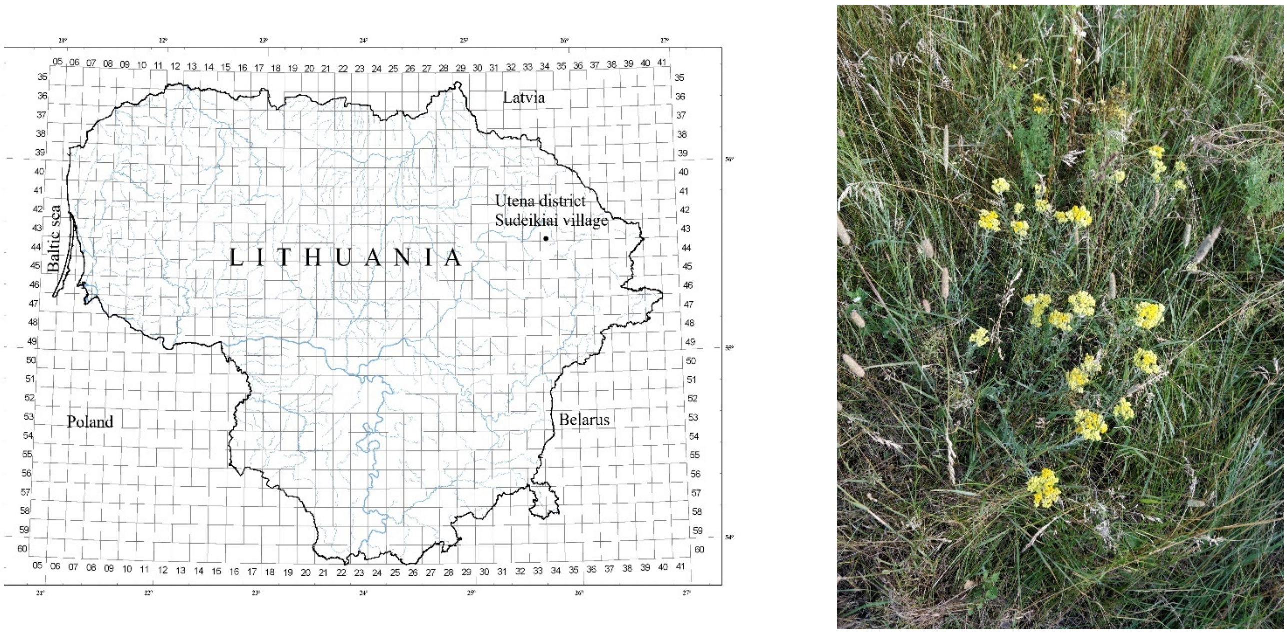

Figure S1. Geographical indication of *H. arenarium* (L.) Moench sampling site in Eastern Lithuania (Utena district).

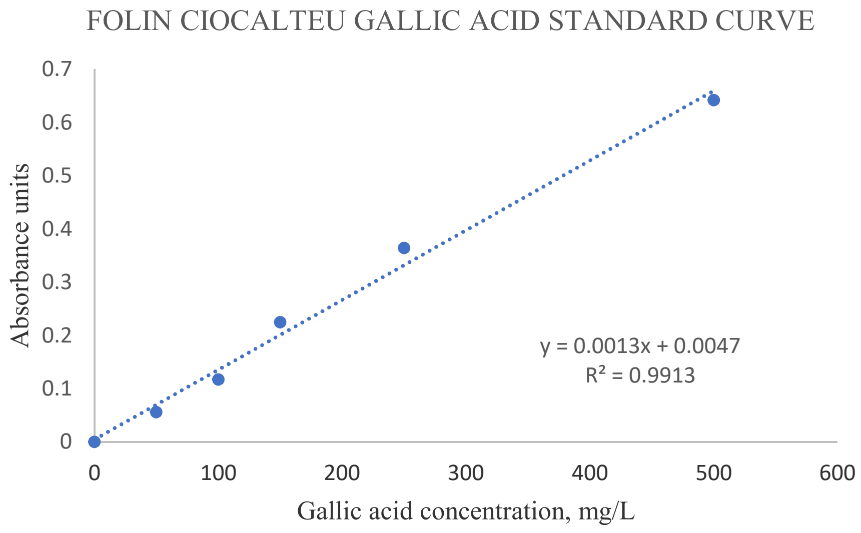

Figure S2. Gallic acid standard calibration curve.

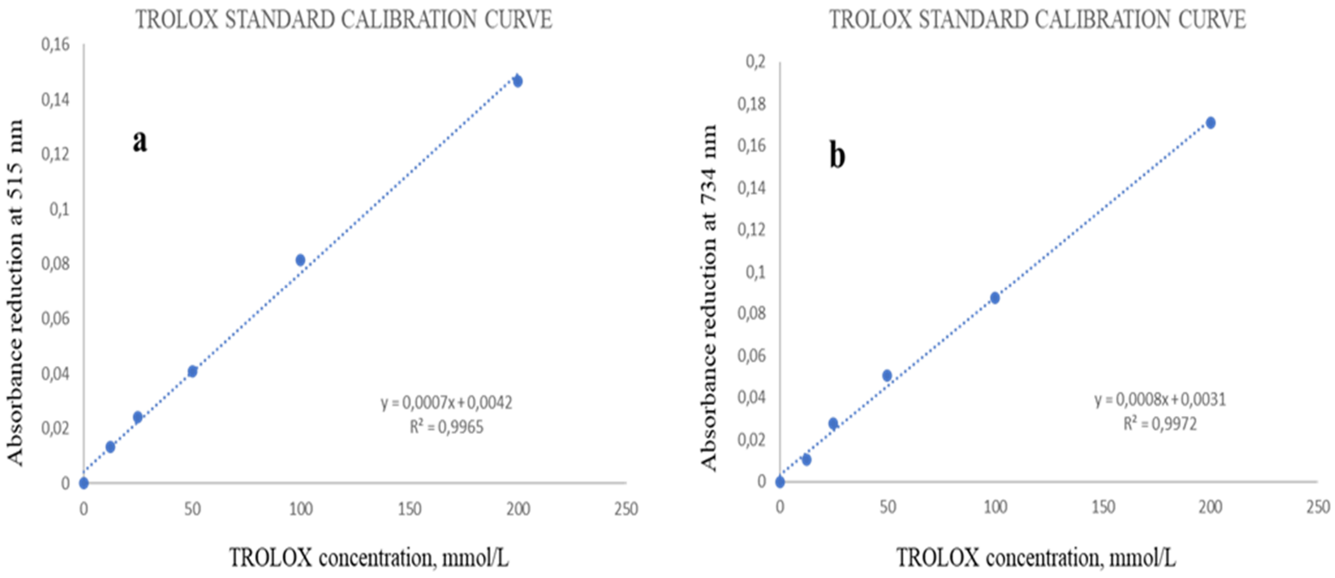

Figure S3. TROLOX standard calibration curves: (**a**) DPPH˙ assay; (**b**) ABTS˙⁺ assay.

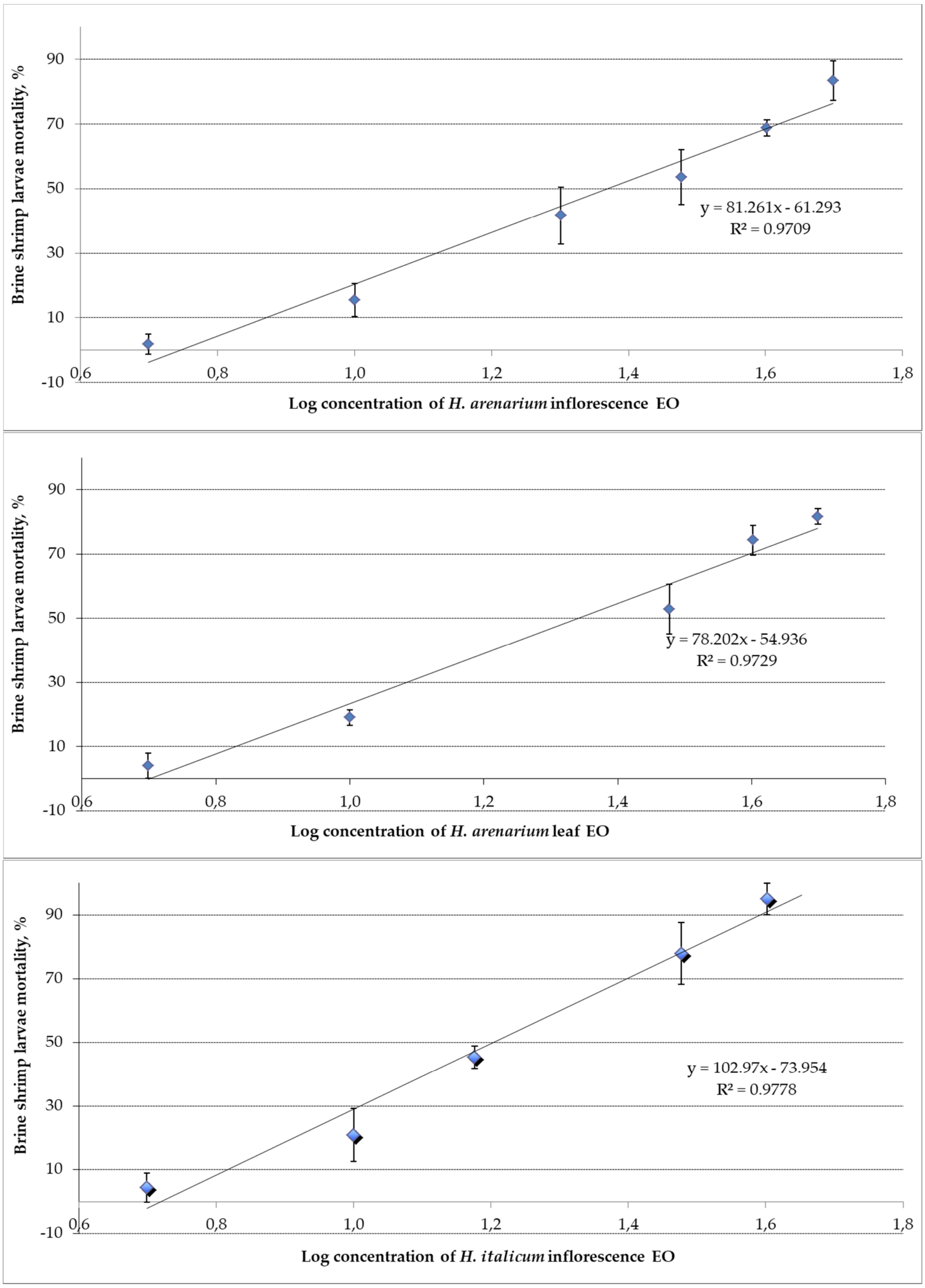

Figure S4. Dependence of brine shrimp *(Artemia salina)* larvae lethality (%) on Log of essential oil concentration in saline water.
